# Ostéogénèse imparfaite létale: diagnostic antenatal

**DOI:** 10.11604/pamj.2016.25.88.5871

**Published:** 2016-10-17

**Authors:** Houda El Mhabrech, Ahmed Zrig, Ines Mazhoud, Leila Njim, Aouatef Hajjeji, Raja Faleh, Ch Hafsa

**Affiliations:** 1 Service d'Imagerie Médicale B, Centre de Maternité et de Néonatologie de Monastir, Université de Monastir, CHU Fattouma Bourguiba Monastir, Tunisie; 2 Service d'Imagerie Médicale A, Université de Monastir, CHU Fattouma Bourguiba Monastir, Tunisie; 3 Service d'Anatomopathologie, Université de Monastir- CHU Fattouma Bourguiba Monastir, Tunisie; 4 Service de Gynécologie-Obstétrique, Centre de Maternité et de Néonatologie de Monastir, Université de Monastir, CHU Fattouma Bourguiba, Monastir, Tunisie

**Keywords:** Ostéogenèse imparfaite léthale, diagnostic anténatal, échographie, TDM, radiographie du squelette fœtal, pathologie, Lethal osteogenesis imperfecta, antenatal diagnosis, ultrasound, fetal, skeletal CT scan, Pathology

## Abstract

L'ostéogenèse imparfaite (OI) est un groupe hétérogène de maladies affectant le collagène de type I et caractérisées par une fragilité osseuse. Les formes létales sont rares et se caractérisent par une micromélie avec déformation des membres. Un diagnostic anténatal d'OI létale a été fait dans deux cas, par échographie à 17 et à 25 semaines d'aménorrhée, complélées par un scanner du squelette fœtal dans un cas. Une interruption thérapeutique de grossesse a été indiquée dans les deux cas.

## Abstract (en)

Osteogenesis imperfecta (OI) is a heterogeneous group of diseases affecting type I collagen and characterized by bone fragility. Lethal forms are rare and are characterized by micromelia associated with limb deformities. We report two cases of prenatally diagnosed lethal OI. Patients underwent ultrasound examination at 17 and 25 weeks of amenorrhoea, supplemented with fetal skeletal CT scanning in one case. Therapeutic abortion was recommended in both cases.

## Introduction

L'ostéogenèse imparfaite (OI) est une maladie génétique caractérisée par le défaut de synthèse quantitatif et/ou qualitatif du collagène de type I, entraînant une fragilité osseuse responsable de déformations osseuses et de fractures multiples. Parmi les nouvelles approches de diagnostic prénatal, l'échographie est une méthode de choix pour montrer les formes pour lesquelles le diagnostic précoce a le plus d'intérêt. Nous rapportons deux cas d'OI diagnostiqués au deuxième trimestre de grossesse par échographie complétée par une TDM du contenu utérin et une radiographie du squelette fœtal. En post natal, le diagnostic a été confirmé par une étude anatomopathologique.

## Patient et observation

### Observation 1

**Figure 1:**
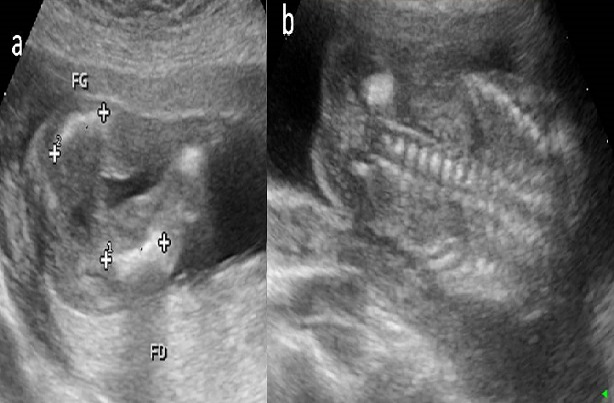
Echographie anténatale à 17 SA: (a) fémur court et incurvé (flèche) (b) aspect aplatit des corps vertébraux (tête de flèche)

**Figure 2:**
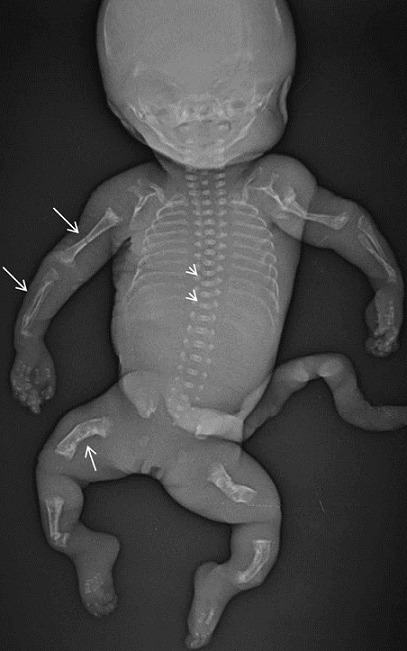
Radiographie du squelette fœtal: fractures multiples des os longs avec déformation des fémurs en receveur téléphonique en rapport aux cals osseux (flèche blanche). Corps vertébraux aplatis (tête de flèche)

Patiente âgée de 27 ans, quatrième geste avait deux enfants vivants en bonne santé et ayant eu une interruption de grossesse pour fémur court et incurvé. Une première échographie réalisait à 8 semaines d'aménorrhée avait montré une grossesse mono embryonnaire évolutive. La deuxième échographie réalisée à 17 semaines d'aménorrhée, avait montré un fœtus en présentation céphalique, mobile et un rythme cardiaque perçu et régulier. Au niveau céphalique, l'analyse des structures cérébrales était facilitée par l'hyper transparence sonore du crâne (Figure 1 a). Les vertèbres étaient petites, hypoplasiques et peu échogènes. Les côtes peu échogènes, déformées, étaient suspectes de fracture. Les membres étaient courts, déformés, avec de multiples fractures rendant les mesures très difficiles (Figure 1 b). Le liquide amniotique était de volume normal et le placenta était sans particularité. Le diagnostic d'OI de type létal était évoqué et une interruption thérapeutique de grossesse était pratiquée. L'examen trouvait un fœtus de sexe féminin; les quatre membres étaient courts et incurvés, le crâne était mou à la palpation sans dysmorphie faciale. La radiographie de squelette montrait un défaut majeur de l'ossification touchant les os longs, le crâne et les corps vertébraux, les côtes ayant l'aspect en tige de bambou avec aspect incurvé des os long vers l'intérieur qui étaient le siège de fractures multiples (Figure 2). L'examen anatomo-pathologique en post mortem objectivait des déformations des membres inférieurs et supérieurs avec incurvation des deux jambes et des deux avant bars. Il n'y avait pas de dysmorphie faciale ni des malformations des mains et des pieds. Ce tableau était en faveur d'une ostéogenèse imparfaite type IIB, C.

### Observation 2

**Figure 3:**
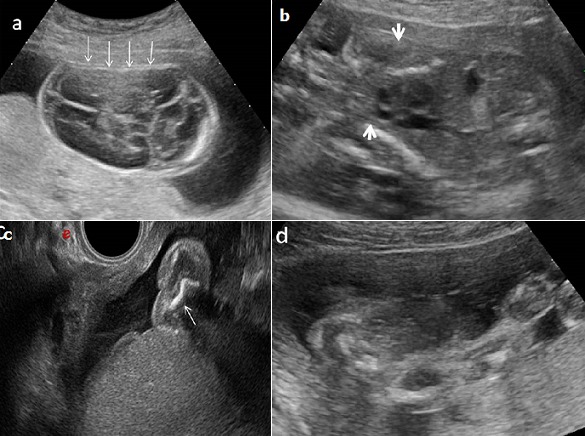
Echographie anténatale à 25 SA: (a) hyper-transparence de l'encéphale qui est déformé sous la sonde (flèche), (b) membre supérieur: nanisme micromélique (c) échographie endovaginale: fémur court et incurvé (flèche), (d): thorax étroit (tête de flèche) contrastant avec un abdomen proéminent

**Figure 4:**
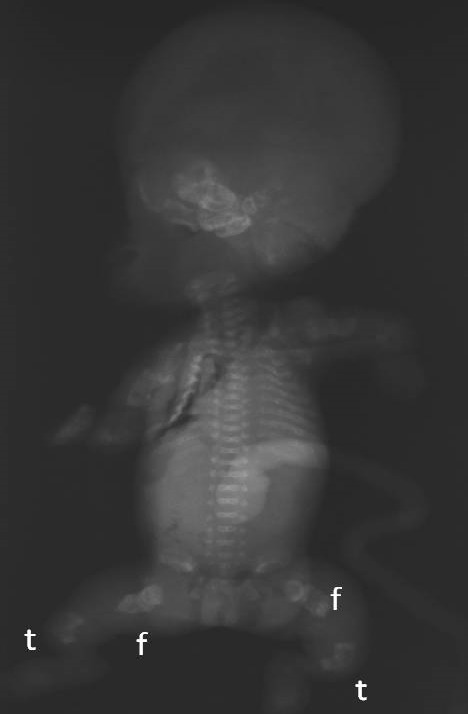
TDM du squelette fœtal (a, b), f: fémur: os long court et épais. Thorax étroit (flèche) associé à une platyspondylie (flèche épaisse) et à un défaut d'ossification du crâne (flèche courbe)

**Figure 5:**
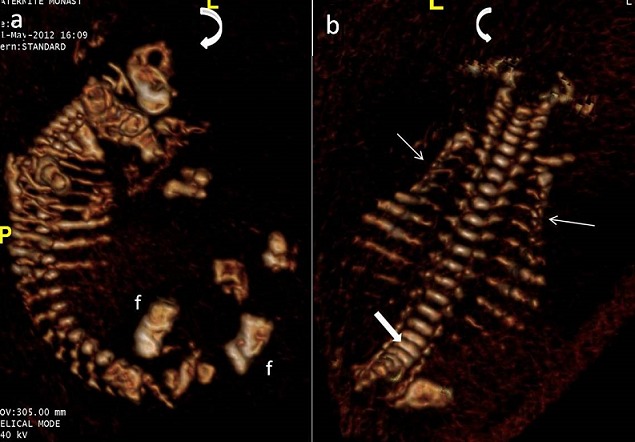
Radiographie du squelette fœtal. f: fémur, t: tibia: défaut d'ossification du crâne, membres très courts et incurvé en receveur téléphonique, côtes déformées porteuses de cals

Patiente de 26 ans, deuxième geste primipare, sans antécédents pathologiques, avait accouché à terme un enfant vivant, en bonne état de santé. La grossesse actuelle était de déroulement normal. L'échographie fœtale réalisée à 25 SA découvrait une grossesse mono fœtale intra-utérine à fœtus vivant. L'étude morphologique retenait un crâne peu ossifiée et dépressible sous la sonde ( Figure 3 a) le thorax était petit et étroit (Figure 3 b). Les membres supérieurs et inférieurs étaient courts et incurvés (Figure 3 c, d). L'hypothèse diagnostique d'une O.I était conforté par une TDM du contenu utérin qui montrant (Figure 4): un fœtus ostéoporotique, des côtes fracturés et enfin des os des membres qui étaient courts, déformés et également fracturés. Les corps vertébraux étaient aplatis Le diagnostic d'OI de type létal a été alors retenu et une interruption thérapeutique de grossesse était pratiquée avec expulsion d'un mort né macéré de sexe féminin de 350 g. Le fœtus avait un crane mou, des oreilles bas implantés associés à un micro-rétrognatisme. Les membres inférieurs et supérieurs étaient courts et d'aspect irréguliers. L'anus était perméable. Les radiographies du squelette (Figure 5) avaient montré un aspect transparent de tout le squelette avec un crane non ossifié. Le rachis avait montré une platispondylie. Les côtes avaient un aspect ondulé en tige de bambou. Les os des membres étaient courts, incurvés et irréguliers. L'examen anatomopathologique a conclu à une ostéochondro-dysplasie létale de type ostéogenèse imparfaite type II.

## Discussion

L'ostéogenèse imparfaite (OI), ou fragilité osseuse constitutionnelle, présente des formes cliniques hétérogènes. L'échographie anténatale permet le diagnostic des formes graves de type II de la classification de Sillence [1–3]. Leurs découvertes se font habituellement lors d'un examen systématique du 2^ème^ trimestre. Parfois, il peut exister un antécédent parental. Une récurrence de forme grave dans la fratrie peut également être observée comme c'est le cas de notre première patiente [1]. A la fin du 1^er^ trimestre, une clarté nucale augmentée, à caryotype normal, peut être observée initialement [2, 4]. Ce signe, non spécifique, augmente le risque de survenue d'une pathologie malformative de nature variable (cœur, appareil digestif, squelette, syndromes géniques,…). Il peut s'observer également dans les autres dysplasies squelettiques. À ce terme, le diagnostic anténatal de forme létale d'OI de type II est possible et évoqué devant l'association pathognomonique de micromélie majeure des 4 membres avec des diaphyses angulées témoignant de fractures et de cals multiples. Une voûte crânienne très peu ossifiée et dépressible à la pression de la sonde est responsable d'une trop bonne visibilité des structures cérébrales [2, 4]. L'analyse fœto-pathologique après interruption médicale de la grossesse proposée, précise le diagnostic en montrant d'une part un nanisme micromélique majeur avec importante déformation des membres, le crâne est mou, le nez est fin avec un rétrognathisme fréquent. L'examen radiographique d'autre part, montre une transparence excessive de l'ensemble du squelette, une ossification de la voûte du crâne quasi absente, des os longs très brefs et déformés, et des côtes en chapelet [2–4]. Au 2^ème^ trimestre, diverses anomalies peuvent être constatées, plus ou moins précocement tel qu'un hydramnios ou un retard de croissance intra utérin. L'analyse morphologique systématique peut objectiver un signe d'alarme important: un fémur court (micromélie) de degré variable [2]. On recherche alors des signes d'orientation diagnostique en faveur de l'OI, car l'éventail des causes de «fémur court » est large. Une diminution de l'ombre acoustique des os longs; des fractures et des cals osseux notamment costales, responsables d'un thorax petit et étroit qui a plutôt une valeur pronostic [2]. En effet, les formes létales type II C de Sillence, s'individualisent des formes II A par des os longs plus minces, en particulier les côtes. Nos observations sont alors classées type IIA de Sillence [1]. L'IRM fœtale dans ce cas peut estimer le volume pulmonaire dont dépend le pronostic vital [5, 6].

Dans les formes avec micromélie majeure, on retrouve les signes associés précédemment décrits en fin de 1er trimestre. Dans ce cas on peut discuter un nanisme thanatophore. Le diagnostic est plus difficile si la micromélie est modérée; devant un fémur court incurvé ou angulé, les signes spécifiques sont souvent peu nets (trop bonne visibilité des structures cérébrales, voûte du crâne dépressible, côtes grêles, caractéristiques du faciès mal identifiables: arête saillante du nez et petit menton, contrastant avec un crâne élargi, …) [2]. Dans nos observations, le contexte fracturaire périnatal est fortement évocateur à lui seul du diagnostic. Seul l'aspect de fémur court et incurvé était présent dans notre premier cas et en l'absence des anomalies costales et cérébrales le diagnostic d'OI était néanmoins suspecté et confirmé en post natal sur les données radiographiques et foeto-pathologiques. Il s'agit donc d'une forme mineure d'OI létale que seule l'échographie anténatale a pu justifier cette conduite. La TDM du squelette fœtal peut dans ces cas difficiles aider au diagnostic en confirmant la dysplasie squelettique et surtout en objectivant les fractures, les cals osseux et les déformations des os long et des côtes [7, 8]. Face à ces cas difficile, on peut discuter un retard de croissance intra-utérin précoce (mosaïques placentaires ou dysgravidie sévère), un nanisme campomélique (incurvation tibiale antérieure médiane) ou une hypophosphatasie (amélioration parfois in utero en fin de grossesse) Une radiographie du « contenu utérin » peut être informative en fin de 2^ème^ et au 3^ème^ trimestre devant le problème diagnostic d'une micromélie modérée [2]. L'évaluation de la « transparence » osseuse en radiographie reste subjective et aléatoire in utero. Avec l'avènement des dernières générations de scanners multidétecteurs, l'approche diagnostique de l'OI létale a nettement progressé [7, 8]. Le scanner fœtal remplace actuellement la radiographie du contenu utérin [7, 8]. L'étude détaillée de l'ensemble du squelette fœtal et plus facile, d'autant plus que la quantité de liquide amniotique, la présentation fœtale et l'obésité maternelle n'influencent pas l'examen, contrairement à l'échographie anténatale [7–9]. L'analyse de la minéralisation osseuse est généralement satisfaisante en scanner multi détecteur à partir de 24 SA [7, 8]. Les fractures et les déformations osseuses sont alors mieux mises en évidence [8]. Selon Miyazaki et All le scanner fœtal confirme le diagnostic d'OI dans tous les cas et redresse le diagnostic échographique dans 59 % des cas [7]. Dans notre deuxième observation cet examen nous a aidé à confirmer le diagnostic.

## Conclusion

En cas de suspicion diagnostique anténatale d'OI de type II, une discussion multi-disciplinaire est nécessaire et éventuellement répétée au vu des arguments supplémentaires donnés par la surveillance in utero (échographie + contenu utérin). Par opposition aux formes létales caricaturales, le diagnostic différentiel est souvent difficile dans les formes sévères avec les autres affections responsables d'une incurvation fémorale (dysplasie campomélique, hypophosphatasie). L'intérêt de coupler alors l'examen échographique à la TDM du contenu utérin est ainsi démontré. Dans ce cas, l'analyse fœto-pathologique est indispensable pour confirmer le diagnostic et étayer le conseil génétique.
